# Shared and Individual Resting-State MEG Network Signatures of Tinnitus Revealed by Holistic Graph Learning

**DOI:** 10.1109/OJEMB.2026.3690604

**Published:** 2026-05-05

**Authors:** Payam S. Shabestari, Harry H. Behjat, Dimitri Van De Ville, Christopher R. Cederroth, Niklas K. Edvall, Adrian Naas, Tobias Kleinjung, Patrick Neff

**Affiliations:** ^1^ Department of Otorhinolaryngology, Head and Neck Surgery, University Hospital ZurichUniversity of Zurich 27217 8091 Zrich Switzerland; ^2^ Department of Clinical Sciences MalmLund University 205 02 Lund Sweden; ^3^ Neuro-X Institute, Ecole Polytechnique Fdrale de Lausanne (EPFL)Campus Biotech CH-1202 Geneva Switzerland; ^4^ Department of Radiology, Medical InformaticsUniversity of Geneva 27212 CH-1211 Geneva Switzerland; ^5^ Laboratory of Translational Auditory Neuroscience, Department of Physiology and PharmacologyKarolinska Institute SE-171 77 Stockholm Sweden; ^6^ Translational Hearing Research, Tbingen Hearing Research Center, Department of Otolaryngology, Head and Neck SurgeryUniversity of Tbingen 72076 Tbingen Germany; ^7^ Bern University of Applied Sciences, Business SchoolInstitute New Work 3005 Bern Switzerland; ^8^ University of FribourgDepartment of Psychology 1700 Fribourg Switzerland; ^9^ Department of Psychiatry, PsychotherapyUniversity of Regensburg 9147 93053 Regensburg Germany

**Keywords:** Brain functional connectivity, fingerprinting, graph learning, magnetoencephalography (MEG), tinnitus

## Abstract

Tinnitus, the perception of sound without an external source, affects many individuals, yet its impact on the brains functional connectome remains underexplored. Traditional functional connectivity (FC) methods, such as Pearson correlation, phase lag index, and coherence, rely on pairwise comparisons between activity of macro-scale brain regions, limiting holistic characterization. We used an approach that estimates the entire connectivity structure by analyzing all time-courses simultaneously, robust even for short recordings and suitable for real-time applications. Using resting-state MEG from tinnitus patients and controls, learned connectomes outperformed correlation-based ones in fingerprinting individuals across test/retest. Group analyses revealed altered FC across multiple frequency bands, impacting default mode, auditory, visual, and salience networks, indicating large-scale reorganization. Tinnitus exhibited highly individualized whole-brain FC profiles, highlighting the importance of individual variability and paving the way for personalized models to optimize patient-specific interventions.

## I. Introduction

Chronic subjective tinnitus is the persistent perception of sound without external sources [1], which can progress into tinnitus disorder, a condition associated with high distress [2]. Tinnitus affects around 14 of the global population, often accompanied by depression and anxiety [3]. With no effective treatments, current approaches mainly alleviate secondary symptoms [4], [5]. Tinnitus commonly arises from hearing loss, causing reduced peripheral input and maladaptive changes in auditory pathways and central networks, which may generate phantom sounds [6]. These changes are reflected in the brains neuroplasticity, including increased gamma and delta and decreased alpha activity in auditory cortical regions [7], [8]. Functional alterations are also observed in resting-state networks, with increased connectivity within and between auditory, default mode, attention, and visual networks [9], [10], [11].

Complex brain functions and neuropathologies like tinnitus rely on anatomically and functionally interconnected networks [12], [13], [14]. Functional connectivity (FC) is commonly used to map these networks via fMRI [15] or M/EEG [16], representing brain regions as vertices and dependenciessuch as Pearson correlation, Phase Lag Index [17], or coherenceas edge weights. Despite its widespread use, FC has key limitations. First, FC may capture spurious correlations from physiological noise [18], particularly in low-SNR regions. Second, FC captures only pairwise relationships, reducing the brains complex dynamics to dyads and limiting the detection of unique network features.

Finn et al. [19] showed that certain FC edges are idiosyncraticdistinctive to each individual and consistent across repeated fMRI sessions. Importantly, these edges, which support individual identification, were also the most predictive of behavioral traits [19]. These findings have spurred research emphasizing individual-specific over generalizable FC patterns, giving rise to the field of brain fingerprinting [20], [21], [22]. More recently, connectome fingerprinting has been extended to other neuroimaging modalities, including MEG [23], [24], broadening its applicability beyond fMRI.

Using principles from graph signal processing [25], [26] and its neuroimaging applications [27], [28], [29], we applied an alternative method to define subject-specific, sparse functional networks [30]. This approach assumes brain activity is a smooth signal on an underlying graph, with neighboring regions showing similar activity [30], [31]. Smoothness, quantified via the graph Laplacian, measures how well observed activity aligns with the network structure. Previous EEG [32], [33] and fMRI [34], [35] studies demonstrated its utility in identifying individualized networks for motor imagery decoding and subject identification. Advantages include: i) reducing spurious correlations from synchronized noise, as each time point is treated independently, and ii) leveraging all time courses simultaneously, overcoming dyadic limitations of traditional pairwise FC and providing a more robust, holistic view of functional connectivity.

We further examined brain connectivity in tinnitus by assessing: i) the robustness of graphs using only a small fraction of time samples, demonstrating efficiency in data-limited scenarios; ii) group-level differences between controls and tinnitus patients, highlighting altered connections in auditory and memory-related regions; iii) within-session connectivity fingerprints, showing consistent individual FC profiles; and iv) the distribution of high-fingerprint connections, revealing key involvement of regions in tinnitus, including the medial posterior cingulate and ventro-medial prefrontal cortex.

## II. Results

### A. Network Robustness

**Figure 1.**
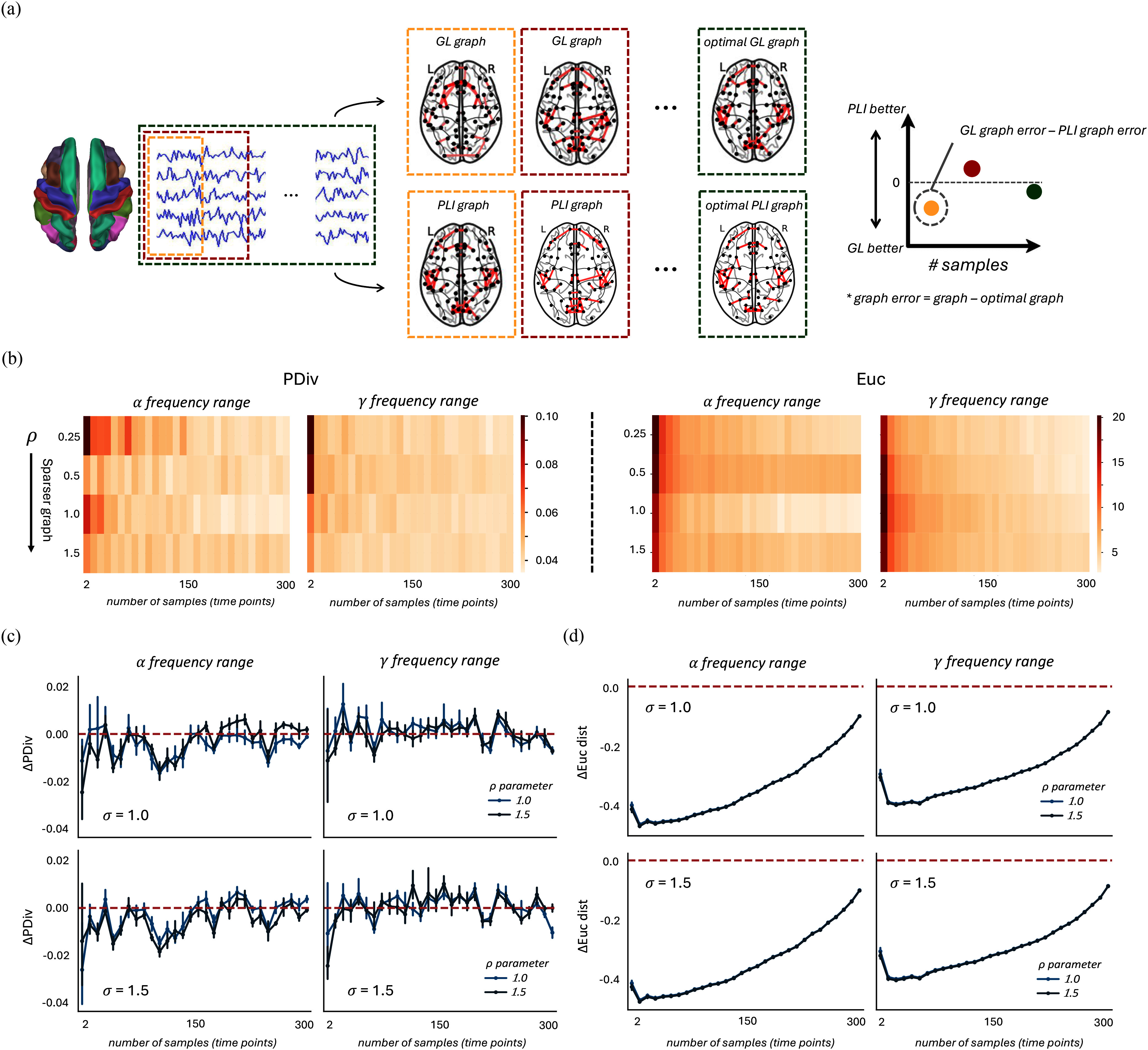
(**a**) Overview of processing steps: graph structures inferred from brain parcel signals across various time windows, and compared to the optimal graph learned from the entire recording. The same analysis was conducted using the PLI method, and the results from both approaches were then compared. (**b**) PDiv and Euclidean distances were calculated between each constructed graph and the optimal graph at alpha and gamma frequency ranges. Differences in PDivs (**c**) and Euclidean distances (**d**) between PLI and GL methods; negative values indicate that the GL method results in less divergence (greater similarity) to the optimal graph compared to the PLI method.

Fig. 1(b) shows portrait divergence (PDiv) and Euclidean distance between each learned graph and the optimal graph across different $\rho$ values ($\sigma =1$). Increasing training samples reduced both metrics, indicating closer alignment with the ideal graph. The absolute values of these metrics for graph learning (GL) based connectivity are provided in Supplementary Figure S4. To benchmark against PLI, we compared PDiv and Euclidean distance from PLI-derived connectomes to graph learning (GL). Figs. 1(c) and (d) show these differences for alpha and gamma bands. Negative $\Delta$PDiv and $\Delta$Euc indicate GL yields lower divergence and distance, reflecting greater similarity to the ideal graph in both local and global structures than PLI. These results demonstrate that graph learning provides more accurate and robust estimates of FC than conventional pairwise methods, particularly in data-limited scenarios.

### B. Statistical Group Comparison of Functional Networks of Tinnitus and Healthy Controls

**Figure 2.**
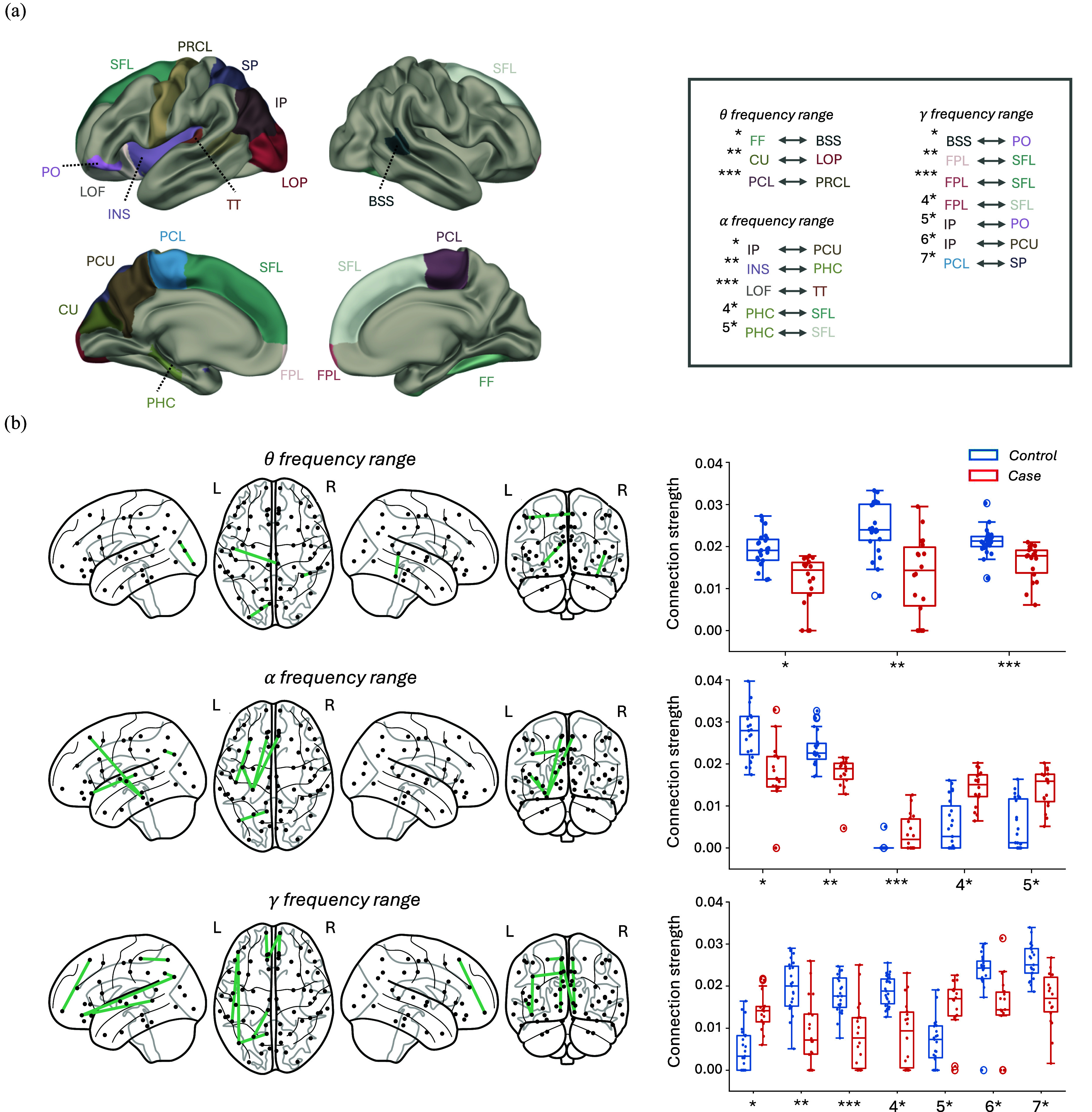
(**a**) The significantly different edges over ROIs (i.e., between centroids of the ROIs) are shown on a semi-inflated standard brain surface (left/right hemispheres, lateral and medial views). Abbreviations not specified above: FF fusiform gyrus, BSS banks of the superior temporal sulcus, CU cuneus, LOP lateral occipital cortex, PCL paracentral lobule, PRCL precentral gyrus, IP inferior parietal lobule, PCU precuneus, INS insula, PHC parahippocampal gyrus, LOF lateral orbitofrontal cortex, TT transverse temporal gyrus, SFL superior frontal gyrus, PO pars orbitalis and FPL frontal pole. (**b**) Significantly different graph edges (${\mathit{p}} < 0.05$) between the two groups are illustrated for the canonical frequency bands (i.e theta, alpha and gamma). On the right, boxplots display the distribution of these connection strengths across subjects for each band in both control (blue) and tinnitus patient (red) groups. Connection IDs (x-axis labels) in the boxplots correspond to those shown in panel (a).

Group-level analysis revealed significant connectivity differences in theta (48 Hz), alpha (813 Hz), and gamma (3080 Hz) bands (Fig. 2), but not delta (0.54 Hz) or beta (1330 Hz). In theta, tinnitus patients showed reduced connectivity between fusiformright superior temporal sulcus ($t(39)=4.65, p=0.042$), left cuneuslateral occipital ($t(39)=4.54, p=0.044$), and paracentralprecentral gyri ($t(39)=4.27, p=0.046$). In alpha, altered connections were found between parahippocampusleft insula ($t(39)=4.74, p=0.039$), bilateral superior frontal gyri (PHC-lh vs SFL-lh: $t(39)=-5.42, p=0.031$; $t(39)=-5.39, p=0.031$), and stronger links from lateral orbitofrontal to transverse temporal gyrus ($t(39)=-3.68, p=0.046$), suggesting increased alpha connectivity in memoryfrontal networks. In gamma, controls showed stronger superior frontalfrontal pole connectivity (SFL-rh vs FP-rh: $t(39)=5.51, p=0.035$; SFL-lh vs FP-rh: $t(39)=4.88, p=0.040$; SFL-lh vs FP-lh: $t(39)=4.75, p=0.041$), while tinnitus patients had enhanced connections among inferior parietal, precuneus ($t(39)=4.0, p=0.045$), pars orbitalis ($t(39)=-4.71, p=0.041$), superior parietalparacentral ($t(39)=3.47, p=0.046$), and superior temporal sulcus ($t(39)=-6.03, p=0.029$), implicating attentionsensory networks. Together, these findings show that tinnitus is associated with frequency-specific alterations in large-scale brain networks, involving auditory, default mode, and attention-related regions. Notably, our results reveal both increases and decreases in connectivity, suggesting a more complex pattern of network reorganization than previously reported [36]. PTA correlations are in Supplementary Figure S1.

### C. Within-Session Functional Network Fingerprints

**Figure 3.**
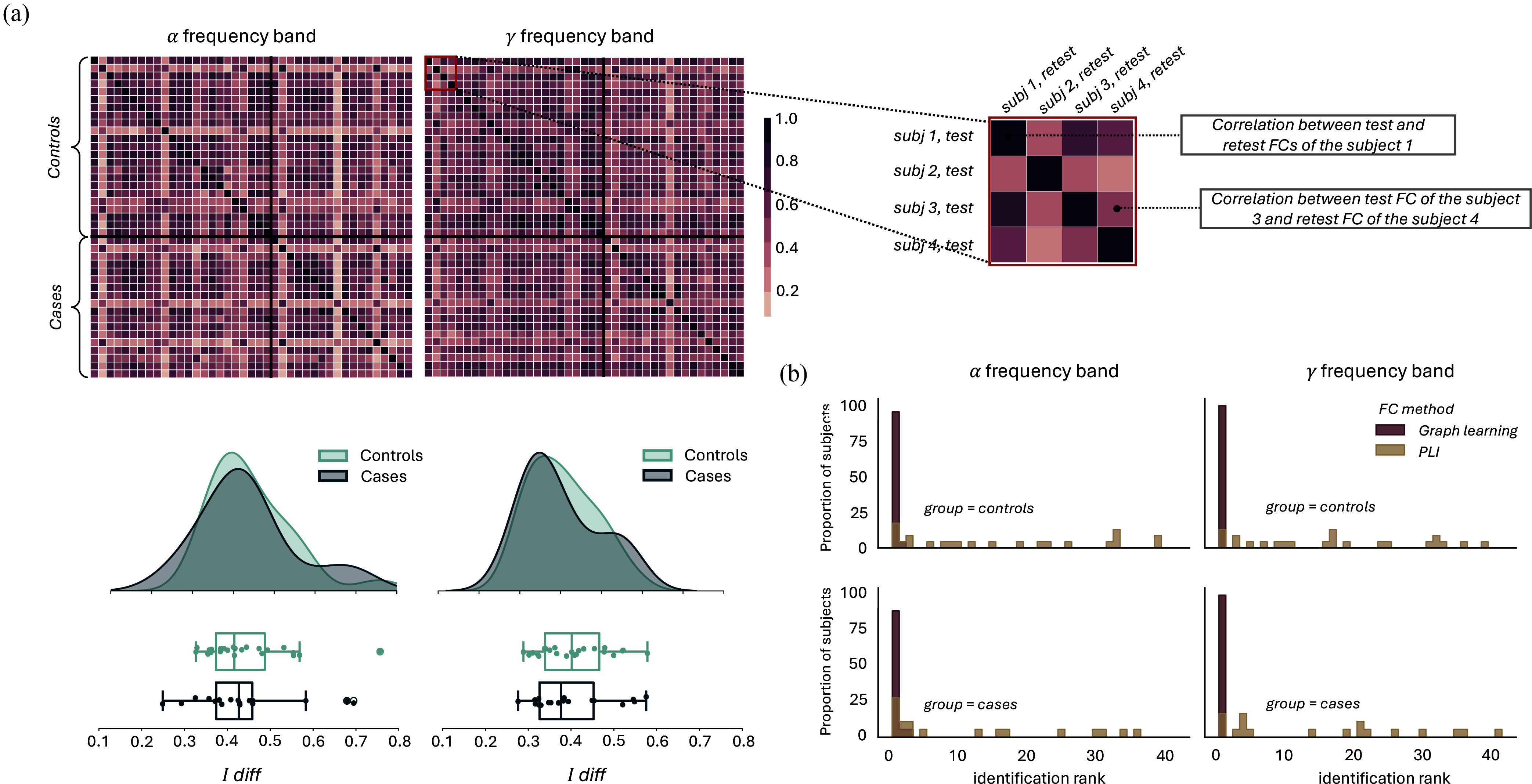
(**a**) The identifiability matrix shows within-subjects similarity (Iself, diagonal elements) and between-subjects similarity (off-diagonal elements) across all subjects (tinnitus + control) for frequency bands alpha and gamma. The density and box plots of Idiff values are shown below. The Idiff value for each subject is calculated as the difference between their Iself value and the average of their off-diagonal values, highlighting the subjects distinctiveness in functional connectivity. (**b**) Bar plots showing the identification ranks in two groups (tinnitus and control) derived via the GL and PLI methods, comparing the test and retest FCs among subjects.

**Figure 4.**
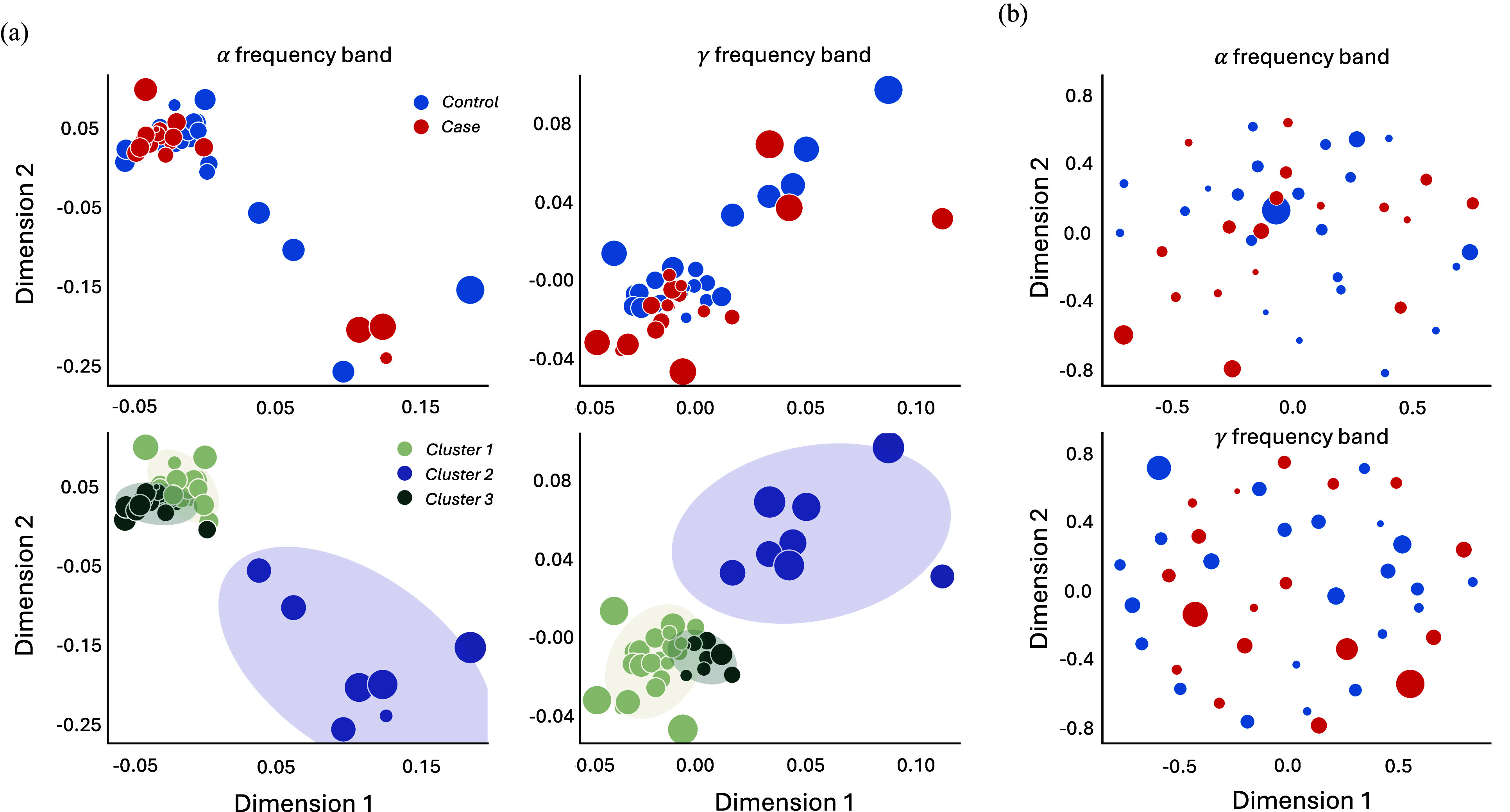
(**a**) The 2D MDS representation of the similarity matrix, derived from the identifiability matrix for the alpha and gamma frequency bands, shows a clear grouping of controls and tinnitus patients. Below this, clustering results into three distinct clusters further highlight a more robust group formation compared to the PLI-based fingerprinting as shown in (**b**). The silhouette scores for alpha and gamma band clustering are 0.210 and 0.202, respectively. In both panels, the marker sizes correspond to individuals Idiff values, where larger marker sizes indicate greater brain discriminability from others.

Fig. 3 summarizes the subject-specific identifiability of functional connectivity (FC) patterns across alpha and gamma frequency bands (additional bands are shown in Supplementary Figure S2). Fingerprints were assessed in 41 participants (controls: 23, tinnitus: 18) using testretest FC estimates and identifiability analysis. The identifiability matrices (Fig. 3(a)) show a clear dominance of diagonal elements ($I_{self}$) over off-diagonal values, indicating that within-subject similarity exceeds between-subject similarity and that individual FC patterns are both reproducible and distinctive. This is further supported by the distribution of $I_{diff}$, defined as the difference between $I_{self}$ and the average off-diagonal similarity, which quantifies the degree of subject-specific separability. Consistent with this, identification rank analysis (Fig. 3(b) ) demonstrates that graph learning (GL) outperforms Phase Lag Index (PLI)-based connectivity across both frequency bands and groups, ranking first for most subjects ($\sigma,\rho =1$). This indicates that GL more effectively captures individual-specific connectivity patterns compared to conventional pairwise approaches. To further examine the structure of inter-individual variability, we visualized the similarity relationships using multidimensional scaling (MDS). The resulting embedding (Fig. 4(a)) reveals a partial separation between tinnitus patients and controls, suggesting that group-level differences are preserved within the individual similarity structure. Marker size, proportional to $I_{diff}$, indicates that individuals with higher discriminability occupy more distinct positions in this space, and clustering further supports the presence of structured group organization. In contrast, the PLI-based representation (Fig. 4(b)) shows a more diffuse distribution without clear group separation, indicating a reduced ability to capture both individual-specific and group-level organization.

### D. Spatial Specificity of Functional Network Fingerprints in Tinnitus

**Figure 5.**
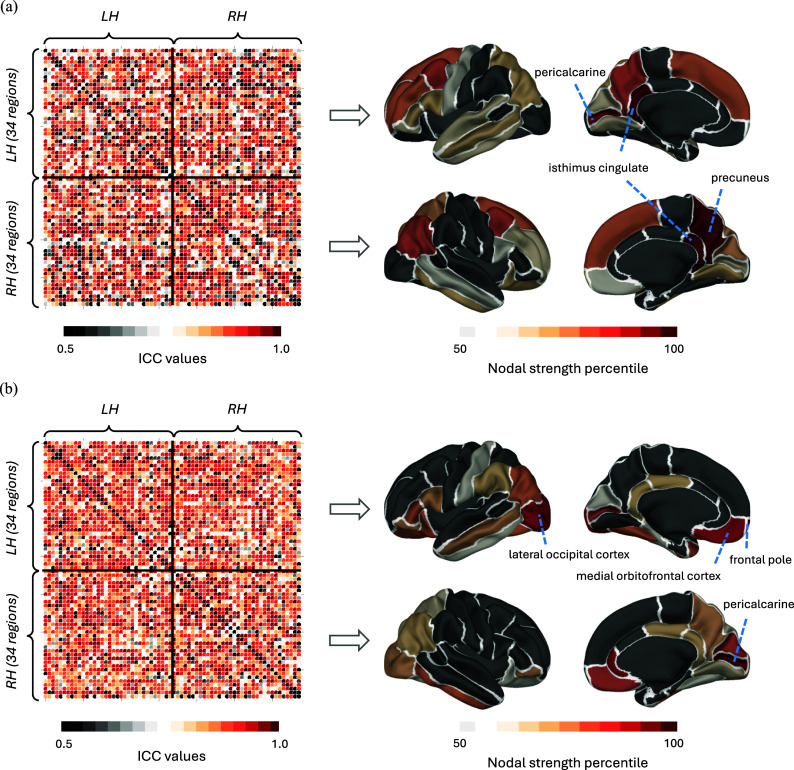
The spatial specificity of functional connectivity (FC) fingerprints in the tinnitus group was assessed using intra-class correlation (ICC), which quantified the fingerprint of each brain edge (connection) for the (**a**) alpha and (**b**) gamma frequency bands. Fingerprinting hubs were identified by calculating the nodal strength of the ICC matrix for each frequency band. Regions with nodal strength in the top 50th percentile were visualized on the cortical surface, highlighting the key hubs contributing to the altered connectivity patterns. In the alpha band (**a**), the highest nodal strength was observed in the left and right isthmus cingulate, precuneus (right hemisphere), and pericalcarine (left hemisphere) cortex. In the gamma band (**b**), the dominant hubs included the left frontal pole, right pericalcarine, left lateral occipital, and left medial orbitofrontal cortex.

We next identified brain regions contributing most to tinnitus variability using edgewise ICC (Section V). Alpha and gamma bands showed reconfiguration of highly identifiable edges (Fig. 5(a) and (b), left). Nodal strength (sum of ICC per region) highlighted top 50th percentile nodes on cortical surfaces (Fig. 5(a) and (b), right). In alpha, key discriminative regions included bilateral isthmus cingulate, right precuneus (DMN), and left pericalcarine. In gamma, regions included left frontal pole, right pericalcarine, and left medial orbitofrontal cortex. These regions appear central to altered tinnitus connectivity. These findings identify specific brain regions contributing to individual variability in tinnitus, highlighting a spatial reorganization of connectivity patterns that may underlie the heterogeneity of the condition. Control spatial maps are shown in Supplementary Figure S3.

## III. Discussion

In this study, we pursue four key objectives: i) to evaluate whether graph learning enables robust estimation of functional connectomes, including in shorter MEG recordings, ii) to quantify frequency-specific group differences in functional connectivity between tinnitus patients and controls, iii) to assess whether graph learning improves individual identifiability of functional networks compared to conventional methods, and iv) to characterize the spatial distribution of connections contributing to subject-specific connectivity patterns in tinnitus. To test robustness, we compared graphs built from subsets of time samples against full-data graphs using Portrait Divergence (PDiv), which quantifies structural similarity (lower values better preservation) [37]. As PDiv assumes unknown node correspondence, we also applied a known node-correspondence metric based on Euclidean edge distances. Both analyses showed stable graphs across sample sizes and parameter settings (Fig. 1(b) ). Comparisons with the Phase Lag Index (PLI) revealed that, while both methods achieved similar PDiv values, graph learning (GL) produced lower distances for smaller sample sizes, often yielding negative PDiv values. Euclidean comparisons confirmed GLs superiority, particularly in data-limited cases (Fig. 1(c) and (d)). This robustness highlights GLs suitability for functional connectivity estimation in task-based designs with short epochs.

Future work should extend this approach by averaging graphs across trials to represent overall connectivity, addressing limitations of conventional methods such as PLI and Coherence, which are constrained by edge effects and timefrequency trade-offs in short signals. The rapid convergence of the GL method highlights its promise for near real-time MEG, where traditional methods struggle with limited data. A key limitation is that GL treats time samples independently, overlooking temporal evolution. Future directions include integrating graph spectral representations of instantaneous MEG data [38] with methods such as the FukunagaKoontz transform [39] to jointly capture spatial and temporal dynamics. While dynamic analyses offer richer insight, static connectivity remains advantageous for simplicity, lower computational demand, and applicability to smaller datasets.

The neural basis of chronic subjective tinnitus is increasingly recognized as an emergent property of distributed large-scale networks rather than isolated auditory lesions. Advances such as graph learning applied to resting-state MEG now allow high-resolution assessment of frequency-specific connectivity changes. Group-level analysis revealed significant alterations in theta, alpha, and gamma connectivity, but not delta or beta (Supplementary Table 1), underscoring their diagnostic relevance and aligning with prior work [10], [11], [40]. Unlike Schlee et al. [11], our alpha-band findings included both decreases and increases: reduced connectivity in medial temporalfrontal circuits of the Default Mode Network (IP, INS, PHC, SFL; Fig. 2) and increased coupling between LOF and TT, linking auditory and salience-related networks. Reduced DMN connectivity supports its hypoactivity in tinnitus [41], [42] and aligns with gating by inhibition models [43], [44]. Enhanced LOFTT coupling suggests hyperactivation of auditorysalience circuits, potentially reflecting compensatory inhibition or pathological focus on internal sound [45]. We also found reduced theta connectivity in tinnitus between fusiform and superior temporal sulcus (right), cuneus and lateral occipital (left), and paracentral lobule and precentral gyrus, implicating visual, sensorimotor, and auditory networks. Our results here align with prior M/EEG and fMRI evidence of large-scale alterations in tinnitus, including widespread non-auditory abnormalities [46], theta-band graph alterations [40], and disrupted DMN and VN connectivity [47]. Increased gamma-band connectivity involving inferior parietal cortex, precuneus, pars orbitalis, and superior temporal sulcus is consistent with earlier reports [46], [48], reflecting heightened sensoryattentional processing and pathological hypersynchrony that sustains tinnitus perception [49].

Synthesizing across bands, tinnitus is marked by gamma hyperconnectivity across auditory, attentional, and limbic networks, alpha hypoactivity in DMN and hyperactivity of auditorysalience networks, and impaired theta integration across sensory networks. These alterations suggest the breakdown of the oscillatory organization that coordinates perception and attention. Our results highlight frequency-specific signatures as mechanistic targets for intervention. Future work should investigate the temporal dynamics of these patterns in different conditions, consider test-retest validation, and translate the insights into neuromodulation strategies [50] to restore network-specific oscillatory balance. Furthermore, the present analysis focuses on connectivity differences at the group- and individual-level, but it does not explicitly account for variability in tinnitus perceptual characteristics, such as sound type (e.g., tonal vs. noise-like), pitch, or loudness. These perceptual features are likely associated with distinct neural mechanisms and may map onto different patterns of network activity. For instance, alterations in auditory cortex connectivity may relate to the spectral characteristics of the perceived sound, whereas changes in salience and default mode networks may reflect attentional capture and the subjective relevance of the percept. Future studies combining detailed perceptual phenotyping with graph-based connectivity analysis could provide a more direct link between tinnitus sound characteristics and large-scale brain network organization.

Our study further demonstrated that individuals can be accurately identified based solely on their brain connectivity profiles. Within-session identification showed reproducible, subject-specific connectivity patterns. Importantly, $I_{self}$ values indicated high testretest consistency across both healthy controls and tinnitus patients, independent of clinical status. Graph learning (GL) improved both global and edgewise fingerprints of functional connectivity compared to a correlation-based method (PLI), achieving higher identification accuracy (Fig. 3(b)). This suggests that GL captures meaningful subject-specific connectivity features rather than shared noise covariance. While group differences in connectivity were detected, substantial across-subject variability underscores the importance of personalized analyses. Spectral clustering of the identifiability similarity matrix revealed high inter-individual variability in FCs. Individuals with high $I_{diff}$ values often appeared as outliers in the MDS space, artificially inflating silhouette scores and limiting clustering cohesion (0.21 for two clusters, 0.202 for three). Low values reflect heterogeneity; our aim was to highlight variability rather than categorical separation. Notably, GL preserved both individual distinctions and group coherence, whereas PLI scattered individuals without clear structure (Fig. 4(b)). It is worth noting that the distribution of $I_{diff}$ values in tinnitus patients (Fig. 3(a)) appears bimodal, in contrast to the more unimodal distribution observed in controls, suggesting a greater heterogeneity in the distinctiveness of individual connectivity profiles under tinnitus. One possible interpretation is the presence of subgroups of patients, with some individuals exhibiting highly distinctive and stable functional connectivity patterns, while others show less separable profiles. Such variability may reflect differences in underlying pathophysiological mechanisms, symptom severity, or compensatory processes. Robust characterization of this inter-individual variabilityand the potential identification of subgroupswill require larger cohorts. This observation nevertheless supports the view that tinnitus is not a unitary condition but rather comprises diverse network-level phenotypes.

The high dimensionality of FC matrices and variability in signal-to-noise ratio limit fine-grained interpretation in small samples; thus, our findings should be viewed as preliminary evidence motivating larger-scale studies. Finally, our results highlight the need for approaches that move beyond casecontrol contrasts to capture individual-level deviations. Normative modeling [51] offers a promising framework by defining typical brain function and quantifying departures at the subject level. Integrating such models with graph-based connectivity estimation could provide deeper insights into heterogeneity in tinnitus and related disorders, supporting a shift toward personalized neuroscience.

Examining the topological distribution of connections underlying individual-specific connectivity patterns, we observed a spatial reconfiguration of regions with the strongest idiosyncratic fingerprints in the tinnitus group (Fig. 5(a), (b), left panels). These regions may represent areas least affected by tinnitus, as they remain distinct across individuals rather than converging toward a shared pattern. To pinpoint regions more directly impacted by tinnitus, we compared their spatial specificity with that of controls (Supplementary Fig. S3). In the alpha band, these regions include the left and right isthmus of the posterior cingulate gyrus, while in the gamma band, they include the left frontal pole, right pericalcarine cortex, and left medial orbitofrontal cortex. In these areas, connectivity profiles exhibit subject-specific variability that exceeds that observed in controls, consistent with the heterogeneity of tinnitus-related distress (e.g., [42]). Conversely, regions with stronger spatial node strength in controls show reduced discriminative power in tinnitus, presenting more homogeneous connectivity profiles in affected individuals. These regions include the bilateral superior frontal cortex, right temporal pole, and right rostral anterior cingulate cortex in the alpha band, and the right pars orbitalis, right rostral middle frontal cortex, and left superior frontal cortex in the gamma band. Such homogenization may reflect both specific and generalized impacts of tinnitus on large-scale brain network organization.

While the present study focuses on characterizing FC alterations in tinnitus, it does not directly address clinical questions such as disease progression, reversibility, or causal relationships with comorbid conditions. These aspects require longitudinal and interventional studies, particularly across transitions from preclinical to acute and chronic tinnitus, to better assess causal mechanisms. Nevertheless, our findings provide insights into the large-scale network organization underlying tinnitus and highlight substantial inter-individual variability. This heterogeneity may contribute to the variable response to existing treatments and suggests that personalized approaches, such as targeted neuromodulation or neurofeedback, could be beneficial. In this context, methods that capture individual-specific connectivity patterns, such as GL, may offer a valuable framework for future translational research, including more detailed phenotyping through integration with multimodal data and further investigation of temporal dynamics and functional relevance.

## IV. Conclusion

Our study introduces the first application of graph learning to M/EEG-based tinnitus research, addressing a key limitation of conventional functional connectivity approaches that rely on pairwise measures and may fail to capture the full structure of large-scale brain networks. By estimating whole-brain connectivity from all regional time courses simultaneously, our framework provides a holistic representation of functional brain organization. Our results indicate that this approach yields more stable connectivity estimates, including in shorter recordings, and improves the identifiability of individual brain network profiles compared to traditional methods. In addition, we observe frequency-specific alterations in large-scale networks, pointing to distributed changes in auditory, default mode, and attention-related systems. Importantly, the findings highlight substantial inter-individual variability in connectivity profiles, challenging the dominant group-level comparison paradigm and supporting the view of tinnitus as a distributed and heterogeneous network disorder rather than a localized dysfunction. By enabling a more detailed characterization of individual connectivity patterns, the proposed framework provides a basis for future work on personalized diagnostics and targeted neuromodulation strategies. Overall, this work contributes to bridging conventional pairwise connectivity approaches and emerging network-level models, expanding the methodological toolkit for studying complex brain disorders and supporting a shift toward individualized, connectivity-based perspectives.

## V. Materials and Methods

### A. Study Population

The research received ethical approval from the Regional Ethics Committee in Stockholm (*Regionala etikprvningsnmnden*, Dnr:2019-05226). All participants provided written informed consent in accordance with the Declaration of Helsinki. Demographic information is shown in Table 1. Details on participant recruitment, MEG and MRI acquisition, and data processing are provided in the Supplementary Information.

**TABLE 1:** Statistical Comparison of the Groups (Controls vs Tinnitus) Before and After Matching

		Before Matching		After Matching	
**Characteristic**		**CO (N23)**	**TI (N18)**	**CO (N18)**	**TI (N18)**
**Age**	Mean $\pm$ SD	28.3 $\pm$ 6.0	36.7 $\pm$ 7.6	30.7 $\pm$ 4.3	36.7 $\pm$ 7.6
	p-value^2^	0.001		0.014	
**Sex**	F, n ()	12 (52)	7 (39)	9 (50)	7 (39)
	M, n ()	11 (48)	11 (61)	9 (50)	11 (61)
	p-value^2^	0.4		0.5	

^1^ *Mean $\pm$ SD; n ()*

^2^ *Wilcoxon rank sum test for age; Pearsons Chi-squared test for sex.*

### B. Construction of Functional Networks

Let $\mathcal {G}= (\mathcal {V}, \mathcal {E}, \mathbf {A})$ denote a weighted, undirected graph, where $\mathcal {V} = \lbrace 1, 2,\ldots, N\rbrace$ represents the graphs finite set of $N$ vertices (nodes), $\mathcal {E}$ denotes the graphs edge set, i.e., pairs $(i, j)$ where $i, j \in \mathcal {V}$, and $\mathbf {A}$ denotes the graphs adjacency matrix, which is symmetric with elements $A_{i,j}=A_{j,i}$ representing the weight of $(i,j)$. In the context of this study, each graph node represents a cortical region of interest (ROI), and a graph signal represents the MEG signal across the entire sets of ROIs at each point of time (i.e. a spatial signal). As such, for each subject, we obtain a set of graph signals, one graph signal per time-point. Thus, the matrix $\mathbf {F}\in \mathbb {R}^{N\times M}$ contains the neural activity recorded across $N$ brain regions over $M$ time samples. The goal of the learning framework is to infer a network (graph) structurewhich is mathematically represented by weighted adjacency matrix $A$wherein the weight of each network edge reflects the strength of activation synchrony between the corresponding brain regions that are connected by the edge. A key feature of this learning approach is that all edge weights are learned jointly from whole-cortex patterns of cortical activity, rather than being derived independently one at a time. The weights in the adjacency matrix indicate the strength of the connection, or similarity between two corresponding vertices, therefore, $A_{i,j}=0$ if there is no connection/similarity between vertices $i$ and $j$. In this work we consider graphs with no self-loops, i.e., $A_{i,i}=0$. The graphs combinatorial Laplacian matrix is defined as:

\begin{align*}
 \mathbf {L}= \mathbf {D}-\mathbf {A}, \tag{1}
\end{align*}

where $\mathbf {D}$ is the diagonal matrix of vertex degrees with its elements given as

\begin{equation*}
 D_{i,i} = \sum _{j} A_{i,j}.
\end{equation*}

Let $\mathbf {f}\in \mathbb {R}^{N}$ denote a graph signal, that is, a real signal defined on the vertices of $\mathcal {G}$ whose $n$-th component ($\mathbf {f}[n]$) represents the signal value at the $n$-th vertex of $\mathcal {G}$. The total variation (TV) of a graph signal $\mathbf {f}$ on graph $\mathcal {G}$ can be quantified using $\mathbf {L}$ as [52]:

\begin{align*}
 \text {TV}(\mathbf {f},\mathbf {L}) = \sum _{(i,j) \in \mathcal {E}} A_{i,j} (\mathbf {f}[i] -\mathbf {f}[j])^{2} = \mathbf {f}^{T} \mathbf {L}\mathbf {f}. \tag{2}
\end{align*}

Larger values of $\text {TV}(\mathbf {f},\mathbf {L})$ indicate greater changes of $\mathbf {f}$ on $\mathcal {G}$, i.e., higher spatial variability, and thus, lower spatial smoothness. Using this notion of smoothness, a sparse graph structure can be inferred from a given set of observations [31]. Let

\begin{equation*}
 \mathbf {F}= [\mathbf {f}_{1}, \mathbf {f}_{2}, \ldots, \mathbf {f}_{M}] \in \mathbb {R}^{N \times M}
\end{equation*}

denote a matrix that stores a set of $M$ measurements on a domain with $N$ elements, and let $\mathbf {Z}$ denote an $N \times N$ matrix with entries that represent the Euclidean distance (or any other distance metric) between pairs of rows in $\mathbf {F}$, i.e.,

\begin{equation*}
 Z_{i,j} = \Vert \mathbf {F}_{i,:} - \mathbf {F}_{j,:} \Vert _{2},
\end{equation*}

where $\mathbf {F}_{k,:}$ denotes the $k$-th row of $\mathbf {F}$, that is measurements from the $k$-th element. A graph structure can be inferred from $\mathbf {F}$ via the optimization [30]:

\begin{align*}
 \min _{\mathbf {A}} \quad & \Vert \mathbf {A}\circ \mathbf {Z} \Vert _{1} - \sigma \mathbf {1}^{T} \log (\mathbf {A}\mathbf {1})+\frac{\rho }{2}\Vert \mathbf {A}\Vert _{F}^{2} \\
 \text {s.t.} \quad & \text {diag}(\mathbf {A}) = 0 \\
 & A_{i,j}=A_{j.i} \geq 0, i\ne j, \tag{3}
\end{align*}

where $\circ$ and $\Vert \cdot \Vert _{F}$ denote the Hadamard product and the Frobenius norm, respectively. $\sigma$ and $\rho$ are regularization parameters; intuitively, smaller values of $\rho$ yield sparser graphs by penalizing edges between vertices with larger $Z_{i,j}$ [30]. The first term in (3) enforces smoothness by invoking that [30]:

\begin{align*}
 0.5 \cdot \Vert \mathbf {A}\circ \mathbf {Z} \Vert _{1} &= \sum _{m=1}^{M} \mathbf {f}_{m}^{T} \mathbf {L}\mathbf {f}_{m}. \tag{4}
\end{align*}

The proposed GL framework relies on the assumption that brain activity can be represented as a smooth signal on an underlying functional network, such that strongly connected brain regions exhibit similar temporal dynamics and therefore smaller pairwise distances. This is consistent with common principles in functional connectivity analysis, where coordinated activity reflects network organization. The model further promotes sparsity, favoring a limited set of meaningful connections to improve interpretability and reduce spurious effects, while enforcing positive node degrees to ensure overall network connectivity. Additionally, time samples are treated as independent observations, enabling robust estimation from short recordings, although temporal dynamics are not explicitly modeled.

To evaluate the performance of the constructed FC graphs, we used the Phase Lag Index (PLI) method. PLI is a reliable measure of phase synchronization that remains unaffected by common sources, such as volume conduction or active reference electrodes. This method is widely utilized to create brain FC networks and it helps in determining the phase relationship between two signals, indicating the extent to which signal leads or lags behind another [17]. Each element of the PLI matrix ($N \times N$) can be quantified as:

\begin{align*}
 PLI_{i,j} = \left| \frac{1}{T} \sum _{t=1}^{T} \operatorname{sign}\left(\Im (S_{ij} \right) \right|, \tag{5}
\end{align*}

where $T$ denotes the total number of time-points and $\Im (S_{ij})$ denotes the imaginary component of the averaged cross-spectral density (CSD) across frequency bins. The CSD is computed between time-courses of two cortical regions $i$ and $j$ (i.e. $\mathbf {F}_{i,:}$ and $\mathbf {F}_{j,:}$) using Morlet wavelets, and it has the dimensions of the number of time points by the number of frequency bins. $PLI_{i,j} = 0$, means that signal $\mathbf {F}_{i,:}$ leads and lags signal $\mathbf {F}_{j,:}$ equally often, while a value greater than 0 means that there is an imbalance in the likelihood for signal $\mathbf {F}_{i,:}$ to be leading or lagging. A value of 1 means that signal $\mathbf {F}_{i,:}$ only leads or only lags signal $\mathbf {F}_{j,:}$.

### C. Network robustness

We evaluated the stability of learned graphs across sample sizes and regularization parameters. Graphs were constructed using four $\sigma$ and $\rho$ values (0.25, 0.5, 1, 1.5) and time windows ranging from 2 to 300 seconds. For each window, data were randomly sampled five times. The graph built from the full 300-second recording was designated the ideal graph ($G_{ideal}$). Similarity was quantified via Portrait divergence (PDiv; [53]), comparing distributions of shortest path lengths, and Euclidean distances between adjacency matrices (upper triangles, 2-norm). Fig. 1(a) illustrates the workflow, where graphs inferred from brain parcels across time windows are compared to the optimal graph.

### D. Statistical group comparison

To compare global cortical connectivity between controls and tinnitus patients, each FC was normalized by its Frobenius norm, with edges $< $1 of the maximum weight excluded. Normality was assessed using the Shapiro-Wilk test (scipy.stats.shapiro); non-normal data were tested with the Mann-Whitney U test (scipy.stats.mannwhitneyu). Bonferroni correction was applied across edges within each frequency band with significance set at $p< 0.05$, and statistical results are reported separately for each band.

### E. Subject identifiability of functional networks

Subject identifiability assesses whether individual connectivity patterns are more self-similar than across subjects. Following [54], we computed an $S \times S$ identifiability matrix $\mathbf {M}$, where $M_{k,l}$ is the Pearson correlation between test (first 2.5 min) and retest (second 2.5 min) FC vectors. Graphs were constructed using the Desikan-Killiany atlas [55], comprising 68 cortical regions. Symmetric graphs allowed use of only the adjacency matrix upper triangles. Identification rank was derived from within-subject correlations, with lower ranks indicating higher identifiability. To explore heterogeneity, we applied spectral clustering (sklearn.cluster.SpectralClustering) to a cosine similarity matrix derived from $\mathbf {M}$, selecting three clusters based on visual inspection and prior expectations.

### F. Spatial specificity of functional network fingerprints

Edgewise intra-class correlation (ICC) was used to quantify test-retest reliability of FC edges, based on a one-way random effects model [56]:

\begin{align*}
 ICC = \frac{MS_{R} - MS_{W}}{MS_{R} + (k - 1)MS_{W}}, \tag{6}
\end{align*}

where $MS_{R}$ is between-subject variance, $MS_{W}$ residual variance, and $k$ the number of sessions. High ICC values indicate stable, subject-specific edges, reflecting reliable network fingerprints. Lower ICC values reflect greater measurement variability. ICC was computed for all edges, identifying connections with high spatial specificity.

## Supplementary Materials

The Supplementary Material provides details on the study population, MEG and MRI acquisition and processing, as well as additional tables and figures supporting the main text.

Supplementary Materials
