# Supplementary material for: Shared and Individual Resting-State MEG Network Signatures of Tinnitus Revealed by Holistic Graph Learning
Source: IEEE Open J Eng Med Biol. 2026 May 5;7:203–13. doi: 10.1109/OJEMB.2026.3690604 (PMC13278749; doi:10.1109/OJEMB.2026.3690604)
Supplement: Supplementary Materials [file supp1-3690604.pdf]

Supplementary Materials

Shared and Individual Resting-State MEG Network Signatures of Tinnitus Revealed by Holistic Graph Learning

Payam S. Shabestari, Harry H. Behjat, *Member, IEEE*, Dimitri Van De Ville, *Fellow, IEEE*, Christopher R. Cederroth, Niklas K. Edvall, Adrian Naas, Tobias Kleinjung, Patrick Neff

I. Study population

THE research received ethical approval from the Regional Ethics Committee in Stockholm, *Regionala etikprövningsnämnden* (Dnr:2019-05226). All participants provided written informed consent after being fully briefed on the study's purpose, scope, and potential risks. The study strictly adhered to the ethical principles of the Declaration of Helsinki. Control individuals consisted of 26 individuals with normal hearing that were recruited via the online platform (accindi.se). Participants with chronic and constant tinnitus (tinnitus always present in silence for $>$ 1 year) were recruited via the accindi platform (n = 8), the Swedish Tinnitus Outreach Project (STOP; n = 4) and the Karolinska Hospital (n = 8). Five participants were excluded due to technical difficulties (MR), claustrophobia and/or low quality signal resulting in a final group size of n = 23 for control group and n = 18 for tinnitus group. Exclusion criteria included pregnancy, sound sensitivity (hyperacusis), psychiatric or neurological conditions, drug use, and non-removable metal implants. Control participants self-reported their sex, resulting in a balanced distribution of males and females, and one identifying as other. Their mean age was 28.4 years (SD ± 5.8), with 88% being right-handed. Tinnitus participants comprised 7 females and 11 males. Their mean age was 36.8 years (SD ± 7.7), with 94.4% being right-handed. We included all available participants and therefore did not enforce strict demographic matching. However, to assess potential confounding effects, we conducted a post-hoc demographic comparison using nearest neighbor matching (matchit R function, MatchIt package). The distribution of sex was relatively balanced and did not change substantially before and after matching. Although a significant difference in age remained between the tinnitus and control groups (ranging from approximately 28 to 36 years), we note that this range falls within a period of stable brain maturation, where major developmental changes are not typically expected. Therefore, we believe it is unlikely that this moderate age difference substantially influenced our connectivity findings. Participants attended the national magnetoencephalography facility (NatMEG) for a single session, where they received detailed procedural information, confirmed the absence or presence of tinnitus or hearing issues, and completed an online survey assessing stress, anxiety, depression, and hyperacusis before undergoing auditory assessments. Resting-state MEG recordings were conducted, followed by a structural MRI scan for each participant using a 3 Tesla GE MR750 Discovery scanner at the MR Center, Karolinska Institutet.

II. MEG and MRI data acquisition

The MEG data consisted of recordings from 18 individuals with tinnitus and 23 controls acquired using a 306-channel Elekta Neuromag184 TRIUX system. Standard MEG preparation procedures were implemented, which included the placement of position indicators (cHPI coils) to monitor movement, 3D registration of the scalp using Polhemus for alignment with structural MRI, and the attachment of electrodes to mitigate muscle artifacts from heart and eye movements. Participants were instructed to sit in a relaxed manner and watch a nature film devoid of sound displayed on a screen in front of them. The silent nature movie was projected onto a screen measuring 72 x 44 cm using an FL35 LED DLP projector positioned outside the magnetically shielded room. The silent movie was used as a neurophysiological "reset" to help participants reach a stable, neutral mental state before the resting-state MEG recording. This minimized variability from prior cognitive or emotional states and reduced anticipatory arousal. It also promoted consistency across participants by establishing a comparable baseline. Importantly, the silent format avoided auditory or language-related stimulation that could affect resting-state activity. The MEG session concluded with a 5-minute resting state recording, during which participants were instructed to maintain a relaxed posture and continue watching the movie as they had done previously during the measurement.
The MRI data consisted of 3D T1-weighted magnetization-prepared rapid gradient-echo (MPRAGE) sequence structural images. These images were acquired using a GE Discovery 3.0T MR scanner, with a voxel size of 1x1x1 mm, a field of view of 256 mm, repetition time (RT) of 2300 ms, and echo time (ET) of 2.98 ms.

III. MEG and MRI data processing pipeline

The MEG data, sampled at 5000 Hz, underwent several preprocessing steps. Signal-Space Projection (SSP) vectors [1] were derived from empty-room recordings conducted before and after the experiment to filter out environmental noise from sources external to the subject and the MEG system. Continuous head position indicator (cHPI) coil signals were used to estimate and track head movements for compensation during recording (see Supplementary Information for a comparison of head movement between the two groups). Automatic identification and correction of noisy and flat MEG channels, along with crosstalk compensation, were performed using spatiotemporal Signal-Space Separation (tSSS) in 10-second intervals [2], [3]. After downsampling to 250 Hz and applying a low-pass filter to prevent aliasing, bandpass filtering between 0.1 and 80

Hz was applied. Independent Component Analysis (ICA) was employed to decompose the data for artifact correction [4]. The minimum number of components capturing at least 95% of data variance was determined. Specifically, for ECG artifacts, a bandpass filter was applied, and relevant components were removed using the Cross-Trial Phase Statistics method [5]. Muscle artifacts were identified and eliminated using the methodology outlined in [6], which involves identifying components with a positive slope in the power spectrum between 7–75 Hz, a peripheral or non-central topographic focus, and low spatial smoothness (i.e., a single, sharply localized activation rather than widespread neural patterns). The signals were bandpass filtered into standard frequency bands: Delta (0.5–4 Hz), Theta (4–8 Hz), Alpha (8–13 Hz), Beta (15–30 Hz), and Gamma (30–80 Hz). This filtering was used to estimate brain activity within each frequency range for further analysis.

We conducted anatomical cortical surface reconstructions of the MRIs using `FreeSurfer` software version 7 [7]. This processing involved cortical surface reconstruction and the generation of source space models. Subsequently, we created Boundary Element Model (BEM) surfaces, encompassing the inner skull, outer skull, and outer skin (scalp), employing the watershed algorithm [3], [8]. A surface-based source space was established, representing bilateral hemispheres, and recursively divided with octahedron spacing. For each subject, a BEM model and its corresponding solution were constructed using the linear collocation method [3]. To ensure accurate alignment, co-registration between the MRI and the head model was performed, initially using three fiducial points provided in the MRI. This alignment was refined through 40 iterations of the Iterative Closest Point (ICP) algorithm [9], with outlier points exceeding a distance of 5 cm excluded and the fitting process reiterated. For each subject, the forward solution was computed utilizing the source model, BEM model, and co-registration data. We computed the noise covariance of the recordings based on empty room recordings. Estimation of noise covariance was carried out using both empirical [3] and shrunk [10] methods, with the best estimator chosen based on log-likelihood and cross-validation with unseen data [11]. Applying the linear minimum-norm inverse method (dSPM) with the noise covariance and forward solution enabled determination of the inverse solution [3], yielding source time courses for each vertex in the source space. Subsequently, a single time course was generated for each brain label by averaging source time courses within vertices located in that specific brain label, irrespective of their orientation. Brain labels and cortical parcellation were derived from the Desikan-Killiany Atlas [12], comprising 68 bi-hemispheric parcels. All MEG processing analysis was performed using `MNE` software (Version 1.6.1).

IV. Comparison of Head Movement Between the Two Groups

We computed framewise displacement (FD) for each subject using the continuous head position indicator (cHPI) traces recorded during MEG acquisition. Specifically, FD at time t was calculated as:

$$\mathrm{FD}_t = |\Delta x_t| + |\Delta y_t| + |\Delta z_t| + r \cdot |\theta_t|$$

which represents the frame-to-frame displacement along the 3 axes. r is radius of the head sphere (commonly assumed to be 50 mm) and θ_t is the rotation angle between consecutive head position matrices, computed as:

$$\theta_t = \cos^{-1}\left(\frac{\mathrm{Tr}(R_{t-1}^{\top} R_t) - 1}{2}\right)$$

A two-sample t-test comparing mean FD between tinnitus patients and control subjects yielded: $t(39) = -0.707, p = 0.483$, indicating no significant difference in head motion between the groups. Furthermore, we would like to emphasize that the MEGIN MaxFilter algorithm already incorporates movement compensation using the cHPI data by realigning the signals to a common head position and removing external interference through Signal Space Separation (SSS). These steps help mitigate potential motion-related confounds in downstream connectivity analysis. Together, these analyses suggest that residual head motion is unlikely to explain the observed group differences in functional connectivity.

References

[1] M. A. Uusitalo and R. J. Ilmoniemi, “Signal-space projection method for separating meg or eeg into components,” *Medical and biological engineering and computing*, vol. 35, pp. 135–140, 1997.
[2] S. Taulu and J. Simola, “Spatiotemporal signal space separation method for rejecting nearby interference in meg measurements,” *Physics in Medicine & Biology*, vol. 51, no. 7, p. 1759, 2006.
[3] A. Gramfort, M. Luessi, E. Larson, D. A. Engemann, D. Strohmeier, C. Brodbeck, R. Goj, M. Jas, T. Brooks, L. Parkkonen *et al.*, “Meg and eeg data analysis with mne-python,” *Frontiers in neuroscience*, p. 267, 2013.
[4] A. J. Bell and T. J. Sejnowski, “An information-maximization approach to blind separation and blind deconvolution,” *Neural computation*, vol. 7, no. 6, pp. 1129–1159, 1995.
[5] J. Dammers, M. Schiek, F. Boers, C. Silex, M. Zvyagintsev, U. Pietrzyk, and K. Mathiak, “Integration of amplitude and phase statistics for complete artifact removal in independent components of neuromagnetic recordings,” *IEEE transactions on biomedical engineering*, vol. 55, no. 10, pp. 2353–2362, 2008.
[6] D. Dharmaprani, H. K. Nguyen, T. W. Lewis, D. DeLosAngeles, J. O. Willoughby, and K. J. Pope, “A comparison of independent component analysis algorithms and measures to discriminate between eeg and artifact components,” in *2016 38th Annual International Conference of the IEEE Engineering in Medicine and Biology Society (EMBC)*. IEEE, 2016, pp. 825–828.
[7] B. Fischl, “Freesurfer,” *Neuroimage*, vol. 62, no. 2, pp. 774–781, 2012.
[8] F. Ségonne, A. M. Dale, E. Busa, M. Glessner, D. Salat, H. K. Hahn, and B. Fischl, “A hybrid approach to the skull stripping problem in mri,” *Neuroimage*, vol. 22, no. 3, pp. 1060–1075, 2004.
[9] Y. Chen and G. Medioni, “Object modelling by registration of multiple range images,” *Image and vision computing*, vol. 10, no. 3, pp. 145–155, 1992.
[10] O. Ledoit and M. Wolf, “A well-conditioned estimator for large-dimensional covariance matrices,” *Journal of multivariate analysis*, vol. 88, no. 2, pp. 365–411, 2004.
[11] D. A. Engemann and A. Gramfort, “Automated model selection in covariance estimation and spatial whitening of meg and eeg signals,” *NeuroImage*, vol. 108, pp. 328–342, 2015.
[12] R. S. Desikan, F. Ségonne, B. Fischl, B. T. Quinn, B. C. Dickerson, D. Blacker, R. L. Buckner, A. M. Dale, R. P. Maguire, B. T. Hyman *et al.*, “An automated labeling system for subdividing the human cerebral cortex on mri scans into gyral based regions of interest,” *Neuroimage*, vol. 31, no. 3, pp. 968–980, 2006.

TABLE I. The effect sizes (Hedges' g) for all connections that showed significant differences between the tinnitus and control groups within specific frequency bands are presented below.

Region 1	Region 2	Frequency band	Effect size
bankssts-rh	fusiform-rh	theta	1.4365
cuneus-lh	lateraloccipital-lh	theta	1.4071
paracentral-rh	precentral-lh	theta	1.3165
inferiorparietal-lh	precuneus-lh	alpha	1.2714
insula-lh	parahippocampal-lh	alpha	1.4618
lateralorbitofrontal-lh	transversetemporal-lh	alpha	-1.1363
parahippocampal-lh	superiorfrontal-lh	alpha	-1.6720
parahippocampal-lh	superiorfrontal-rh	alpha	-1.6627
bankssts-lh	parsorbitalis-lh	gamma	-1.8622
frontalpole-lh	superiorfrontal-lh	gamma	1.4645
frontalpole-rh	superiorfrontal-lh	gamma	1.5075
frontalpole-rh	superiorfrontal-rh	gamma	1.7006
inferiorparietal-lh	parsorbitalis-lh	gamma	-1.4549
inferiorparietal-lh	precuneus-lh	gamma	1.2350
paracentral-lh	superiorparietal-lh	gamma	1.0708

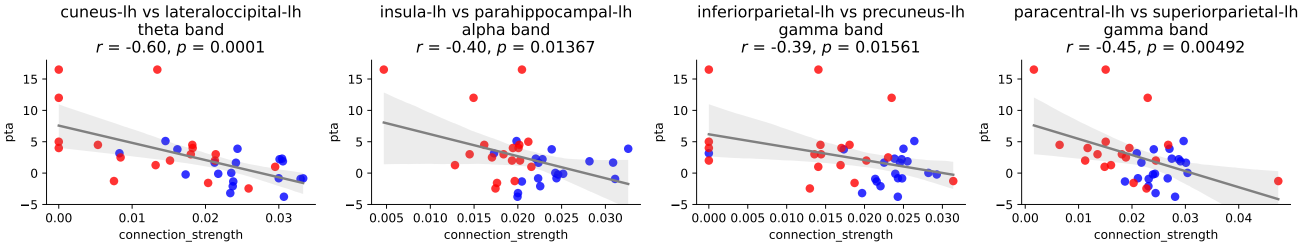

Fig. S1. Correlations between Pure Tone Average (PTA) and the connections that showed statistically significant differences between the two groups are presented. Only connections with significant correlations are shown. Blue dots represent control subjects, and red dots represent individuals with tinnitus.

TABLE II. Descriptive characteristics of the participants. Between parenthesis are either standard deviation (SD) or percentage (%). Abbreviations: Perceived Stress Questionnaire (PSQ), Hyperacusis Questionnaire (HQ), Hospital Anxiety and Depression Score (HADS), Pure Tone Audiometry (PTA), High Frequency (HF), decibel hearing levels (dB HL)

	**Control group (n=23)**	**Tinnitus group (n=18)**
**Age**		
Mean (SD)	29.17 (5.69)	36.83 (7.66)
**Sex**		
Male	11 (47.8%)	11 (61.1%)
Female	11 (47.8%)	7 (38.9%)
Other	1 (4.3%)	0 (0.0%)
**Handedness**		
Right	21 (91.3%)	17 (94.4%)
Left	2 (8.7%)	1 (5.6%)
**Tinnitus**		
Yes, always	0 (0.0%)	18 (100.0%)
Yes, often	0 (0.0%)	0 (0.0%)
Yes, sometimes	0 (0.0%)	0 (0.0%)
Not last year	6 (26.1%)	0 (0.0%)
No, never	16 (69.6%)	0 (0.0%)
Don't know	1 (4.3%)	0 (0.0%)
**Tinnitus lateralization**		
Right	-	1 (5.6%)
Left	-	5 (27.8%)
Both	-	12 (66.7%)
**PSQ score**		
Mean (SD)	0.23 (0.14)	0.41 (0.15)
**HQ score**		
Mean (SD)	9.70 (6.38)	22.61 (8.23)
**HADS Anxiety score**		
Mean (SD)	4.09 (3.12)	8.61 (3.71)
**HADS Depression score**		
Mean (SD)	1.91 (2.63)	4.78 (4.11)
**THI score**		
Mean (SD)	-	43.78 (27.56)
**PTA Left (dBHL)**		
Mean (SD)	3.00 (2.91)	6.85 (6.12)
**HF PTA Left (dBHL)**		
Mean (SD)	8.65 (12.51)	25.59 (22.72)
**PTA Right (dBHL)**		
Mean (SD)	3.87 (3.09)	6.93 (6.44)
**HF PTA Right (dBHL)**		
Mean (SD)	8.54 (9.09)	22.68 (20.80)
**Tin pitch (kHz)**		
Mean (SD)	-	9.94 (7.44)
**Tin loudness (dBHL)**		
Mean (SD)	-	24.35 (21.73)
**Tin loudness (dBSL)**		
Mean (SD)	-	3.38 (8.82)

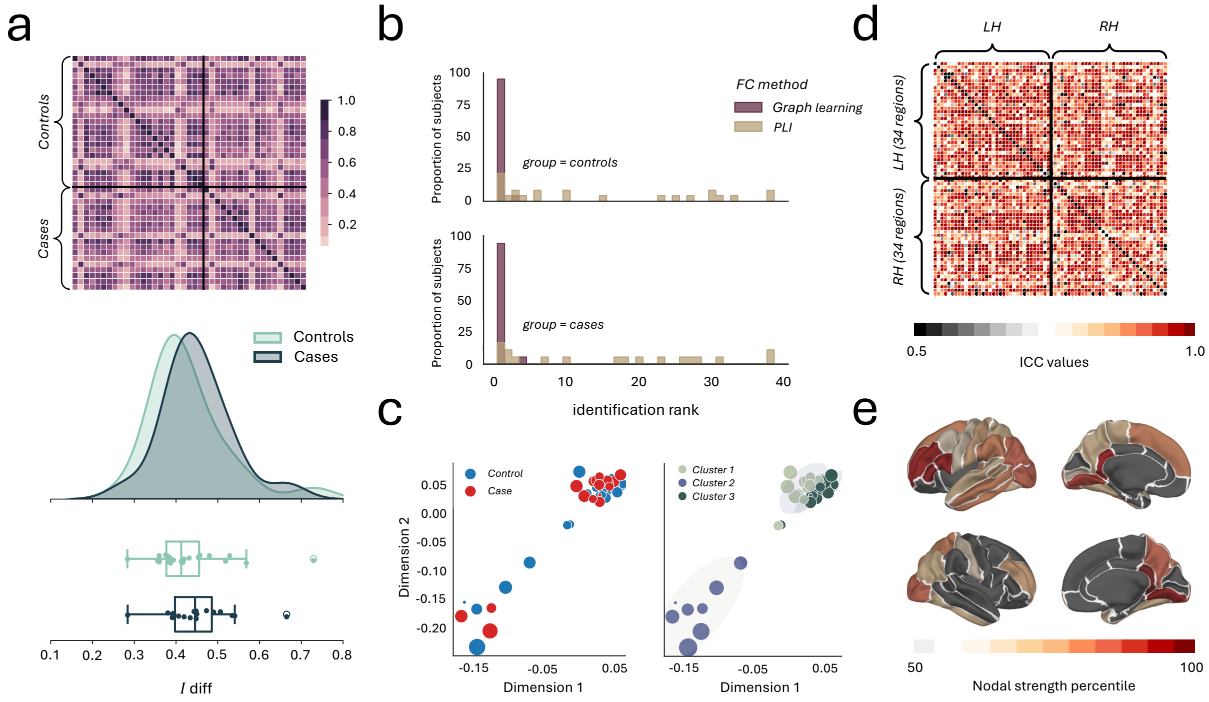

Fig. S2. (**a**) The identifiability matrix shows within-subjects similarity (Iself, diagonal elements) and between-subjects similarity (off-diagonal elements) across all subjects (tinnitus + control) for theta frequency band. The density and box plots of Idiff values are shown below. The Idiff value for each subject is calculated as the difference between their Iself value and the average of their off-diagonal values, highlighting the subject's distinctiveness in functional connectivity. (**b**) Bar plots showing the identification ranks in two groups (tinnitus and control) within theta frequency band derived via the GL and PLI methods, comparing the test and retest FCs among subjects. (**c**) The 2D MDS representation of the similarity matrix, derived from the identifiability matrix for the theta frequency band, shows a clear grouping of controls and tinnitus patients (left panel) together with the clustering results (right panel) (**d**) The spatial specificity of functional connectivity (FC) fingerprints in the tinnitus group was assessed using intra-class correlation (ICC), which quantified the fingerprint of each brain edge (connection) for the theta frequency band. (**e**) Fingerprinting hubs were identified by calculating the nodal strength of the ICC matrix for each frequency band. Regions with nodal strength in the top 50th percentile were visualized on the cortical surface, highlighting the key hubs contributing to the altered connectivity patterns.

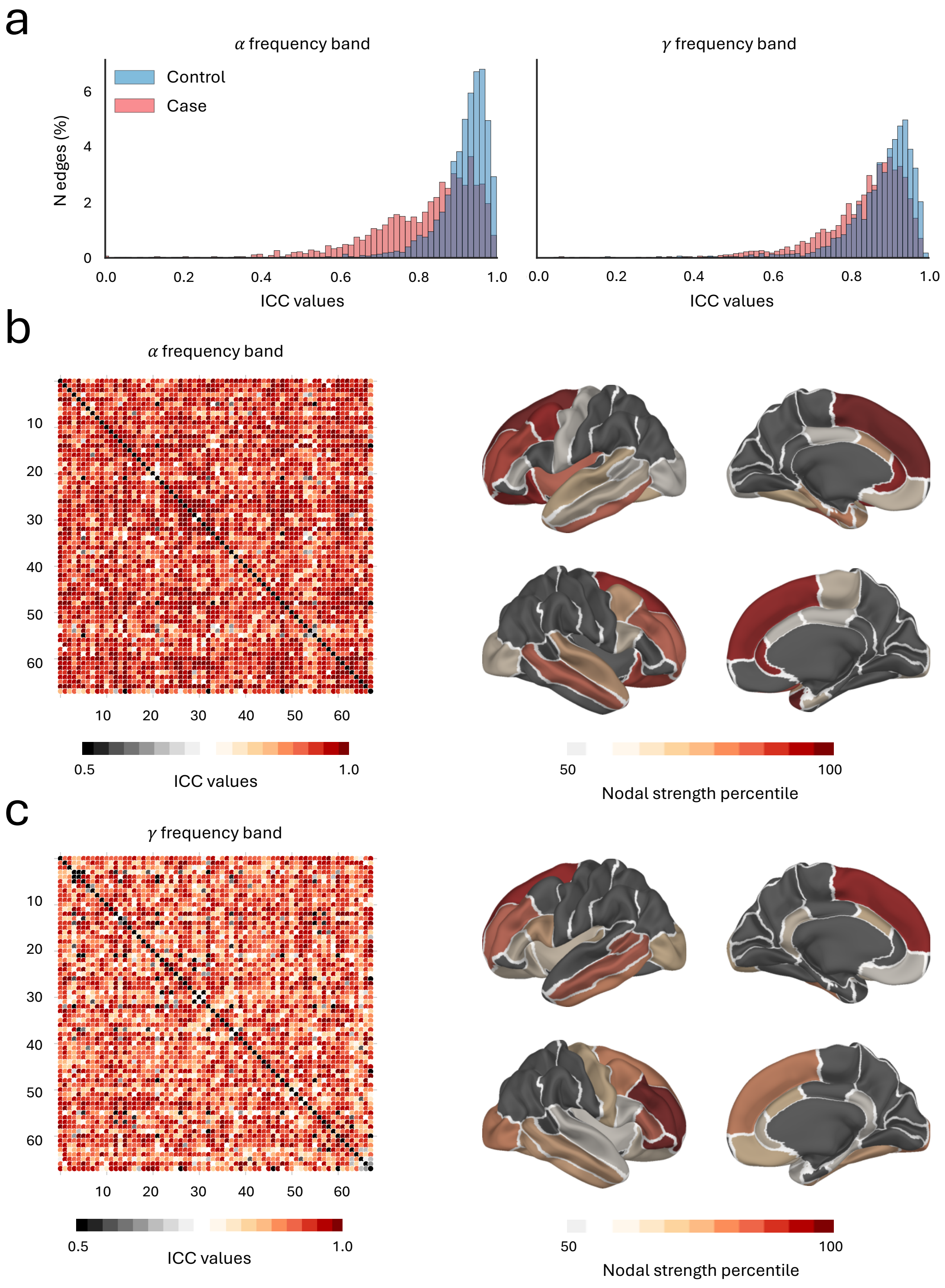

Fig. S3. The spatial specificity of functional connectivity (FC) fingerprints within the control group was evaluated using intra-class correlation (ICC), which measured the importance of each connection in the alpha and gamma frequency bands in discriminating individuals. (**a**) The ICC value distributions for both groups, shown for the alpha band (left panel) and gamma band (right panel), reveal predominantly lower ICC values in the tinnitus group. This pattern suggests greater similarity between functional connectivity within the tinnitus group. (**b**) The nodal strength of the ICC matrix was calculated for each frequency band. Brain regions with nodal strength in the top 25th percentile were mapped onto the cortical surface, revealing the key hubs that contribute to the altered connectivity patterns observed in the control group.

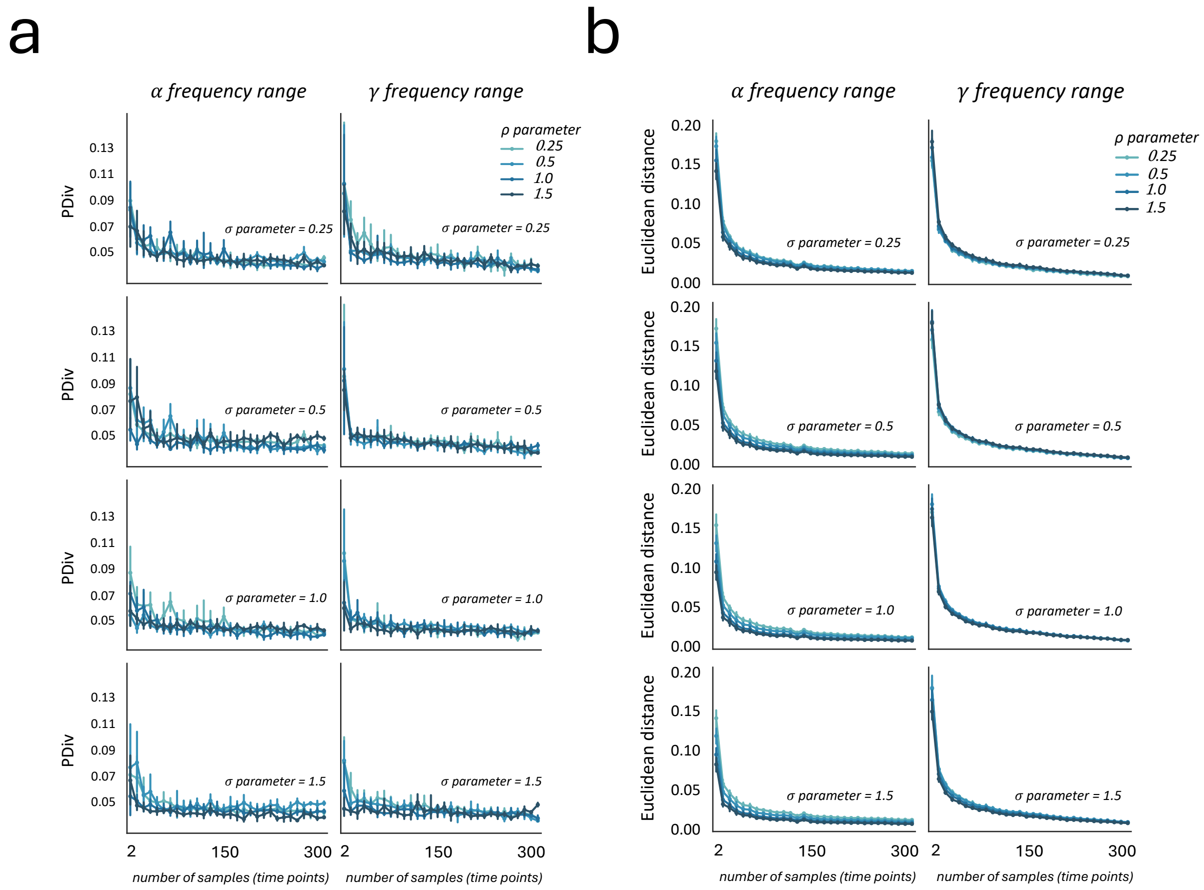

Fig. S4. Graph stability across various regularization parameter configurations and different numbers of time points used for graph learning; PDiv (**a**) and Euclidean distances (**b**) were calculated between each graph and the ideal graph at alpha and gamma frequency ranges.

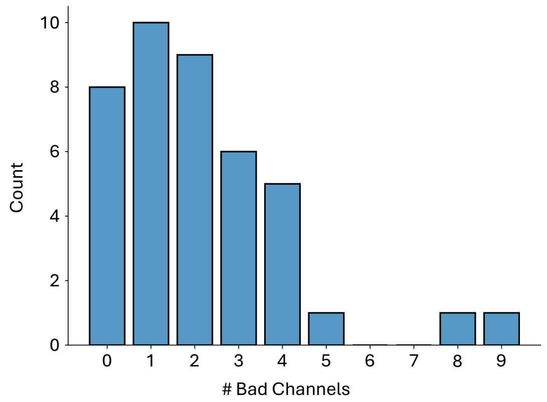

Fig. S5. Histogram showing the number of MEG channels identified as bad (noisy or flat) per recording. Bad channels were automatically annotated and excluded from further analysis. The MEG system includes 102 magnetometers and 204 gradiometers in total.

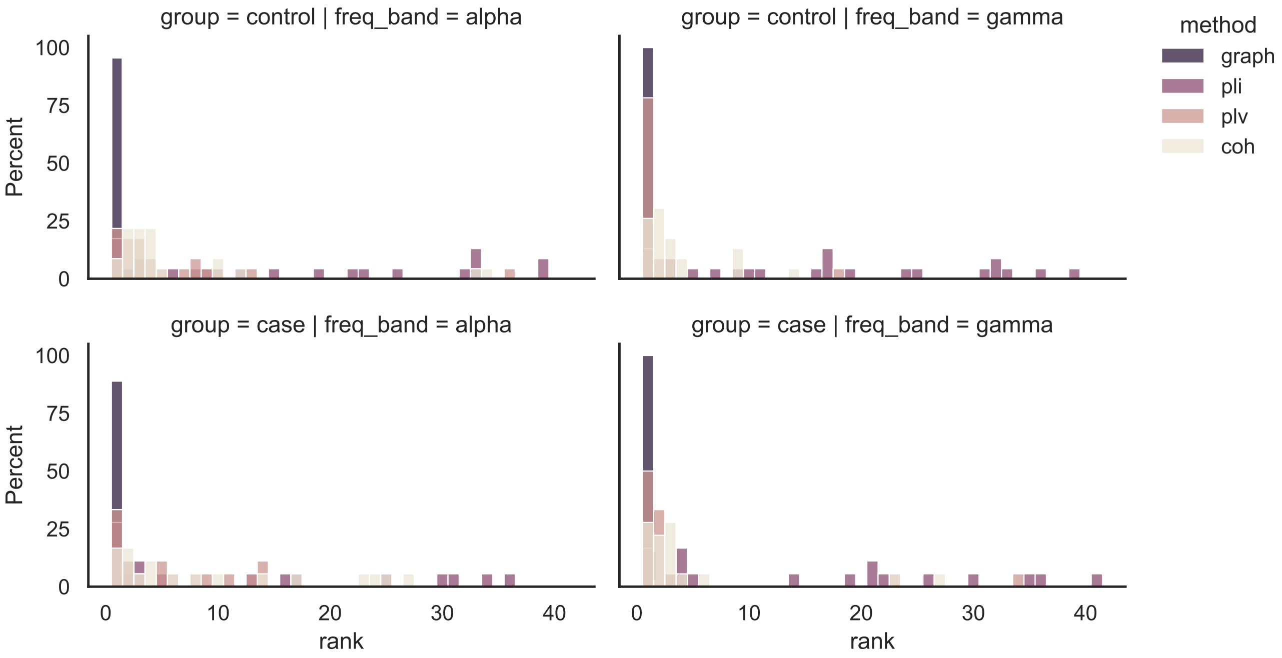

Fig. S6. Bar plots show the identification ranks for the tinnitus and control groups using four different methods: Graph Learning (GL), Phase Lag Index (PLI), Phase Locking Value (PLV), and Coherence (Coh). The comparison is based on test–retest functional connectivity across subjects. It is evident that the GL method outperforms the other three approaches.
